# Cognitive Behavioral Therapy for Insomnia in Postpartum Depression: A Meta-Analysis of Therapeutic Outcomes and Barriers to Clinical Use

**DOI:** 10.7759/cureus.102902

**Published:** 2026-02-03

**Authors:** Zeeshan Ali, Osama Akhtar, Zekra Ehsaan, Muhammad Maaz, Shadman Ahmad, Muhammad Daniyal

**Affiliations:** 1 Psychiatry and Behavioral Sciences, Hayatabad Medical Complex Peshawar, Peshawar, PAK; 2 School of Medicine, Khyber Medical University, Peshawar, PAK; 3 School of Medicine, Bacha Khan Medical College, Mardan, PAK

**Keywords:** cognitive behavioral therapy for insomnia (cbt-i), maternal mental health, perinatal insomnia, postpartum depression, treatment barriers

## Abstract

Cognitive behavioral therapy for insomnia (CBT-I) is the first-line treatment for insomnia in the general population and pregnant women. However, its effects on postpartum depression remain underexplored.

Objectives of this meta-analytic review were to evaluate the effects of CBT-I on postpartum depression, insomnia severity, and total sleep time in perinatal women.

PubMed, ScienceDirect, and Google Scholar databases were searched. Randomized controlled trials with perinatal women, CBT-I as intervention, and comparison with any other intervention were included. Bias was evaluated using the Cochrane Risk of Bias 2 (RoB 2) tool. Four studies were deemed eligible, with a combined sample size of 381 participants. Insomnia severity, postpartum depression, and total sleep time were selected as outcomes. Mean differences were calculated with 95% confidence interval (CI), and heterogeneity was assessed using *I*^2^ statistics.

For insomnia severity, a mean difference of -2.30 (95% CI: -4.10 to -0.49, *P* = 0.0126) was obtained, favoring the CBT-I group. For postpartum depression, a standardized mean difference of -0.20 (95% CI: -0.40 to 0.00; *P* = 0.0502) showed borderline statistical significance, also favoring the CBT-I group. However, for total sleep time, a mean difference of 0.44 (95% CI: -0.04 to 0.92; *P* = 0.075) with 66% heterogeneity showed nonsignificant results, mainly due to an outlier study. With this outlier study, the heterogeneity dropped to 0%, the mean difference increased to 0.68 (95% CI: 0.37-0.99), reaching statistical significance (*P* < 0.0001).

This meta-analysis demonstrates that CBT-I significantly reduces insomnia severity and improves total sleep time in perinatal women, with a borderline significant reduction in postpartum depression scores. Despite its growing evidence, it remains an underutilized treatment option. By incorporating it, the overall quality of maternal healthcare services can be improved.

## Introduction and background

Disturbed sleep is almost a universal experience for new mothers. Research shows that 97% of mothers face sleep problems in the postpartum period [1]. These sleep problems can even extend well beyond the postpartum period. Approximately 50% of the women with insomnia during pregnancy have their symptoms persist into at least two years postpartum. Insomnia is defined as difficulty with sleep initiation, maintenance, or early awakening that results in daytime consequences and functional impairment [2]. For chronic insomnia, symptoms must occur at least three nights per week for at least three months [3]. Short-term insomnia has similar diagnostic criteria but with a duration of less than three months and no specific frequency requirements [4]. Data show a prevalence of 60% during pregnancy and the early postpartum period, and 41% at two years postpartum, which is a critical period for newborn development [5]. The perinatal period is defined by the World Health Organization as the period of pregnancy and the 6 weeks following delivery [6]. Additionally, the postpartum period is described as beginning one hour after delivery of the placenta and continuing until 6 weeks, or 42 days, after delivery [7].

Cognitive behavioral therapy for insomnia (CBT-I) is built on Spielman's behavioral model, commonly known as *the 3P Model*. It identifies three key factors that contribute to chronic insomnia, including predisposing factors (like hyperreactivity and altered circadian rhythms), precipitating factors (such as medical problems or stressors), and lastly, perpetuating factors (such as spending excessive time in bed trying to rest) [8]. Research on CBT-I specifically for pregnant women has gained momentum recently. Several notable randomized controlled trials (RCTs) established the efficacy of CBT-I during pregnancy. In an RCT of 208 pregnant women with insomnia symptoms, participants received either six weekly sessions of CBT-I (approximately 20 minutes each) or standard treatment. Women who were in the CBT-I group underwent statistically significant improvements in their insomnia symptoms compared to those having standard treatment [9].

Furthermore, another RCT on CBT-I for pregnant women showed greater improvements in insomnia severity, total wake time, and Edinburgh Postnatal Depression Scale scores compared to control therapy [10]. Current clinical recommendations prefer non-pharmacological interventions, including CBT-I, as the first-line treatment options for new mothers having sleep disturbances [11]. But there is no direct recommended guideline-backed clinical protocol for including CBT-I as a routine treatment option available for those in the perinatal period.

Comprehensive meta-analyses have established the evidence base for CBT-I in various psychiatric conditions, including depression. A 2022 meta-analysis of 22 RCTs examining CBT-I for patients with comorbid insomnia and mental disorders found that CBT-I produced big improvements for insomnia and reduced the severity of mental disorders [12]. In the perinatal period specifically, a meta-analysis of 40 studies examined the effects of cognitive behavioral therapy during pregnancy and the first year postpartum. This study found that CBT resulted in significant reductions in depressive symptoms [13]. This broader evidence supporting CBT provides a strong rationale for the development and recommendation of more specific CBT-I interventions for pregnant women.

The Diagnostic and Statistical Manual of Mental Disorders, Fifth Edition (DSM-5) defines postpartum depression as an episode of major depression with peripartum onset. It can occur during pregnancy or within the first four weeks postpartum [14]. Several biological and psychological mechanisms have been proposed to explain the strong relationship between poor sleep and postpartum depression, one of which is that poor sleep may lead to circadian rhythm dysregulation. This predisposes individuals to depression [15,16]. Newborns’ care and feeding responsibilities particularly make this circadian disruption unavoidable, as these factors create sleep fragmentation regardless of the time of day or night [17,18]. Studies have established that fatigue, poor sleep quality, and postpartum depression interact synergistically and increase in severity together, creating a self-reinforcing cycle [1].

Despite the growing evidence base for CBT-I in perinatal women, several important literature gaps and future research directions have been identified, with the most important being no meta-analysis available, which usually constitutes the strongest evidence for guideline development to include it as a routine treatment option during the perinatal period. Furthermore, the lack of trained personnel makes it an underutilized clinical service available in maternal health services [19,20]. Therefore, this meta-analysis aims to synthesize the available evidence on the effects of CBT-I on (1) postpartum depression, (2) insomnia severity, and (3) total sleep time in perinatal women.

## Review

Methodology

**Figure 1:**
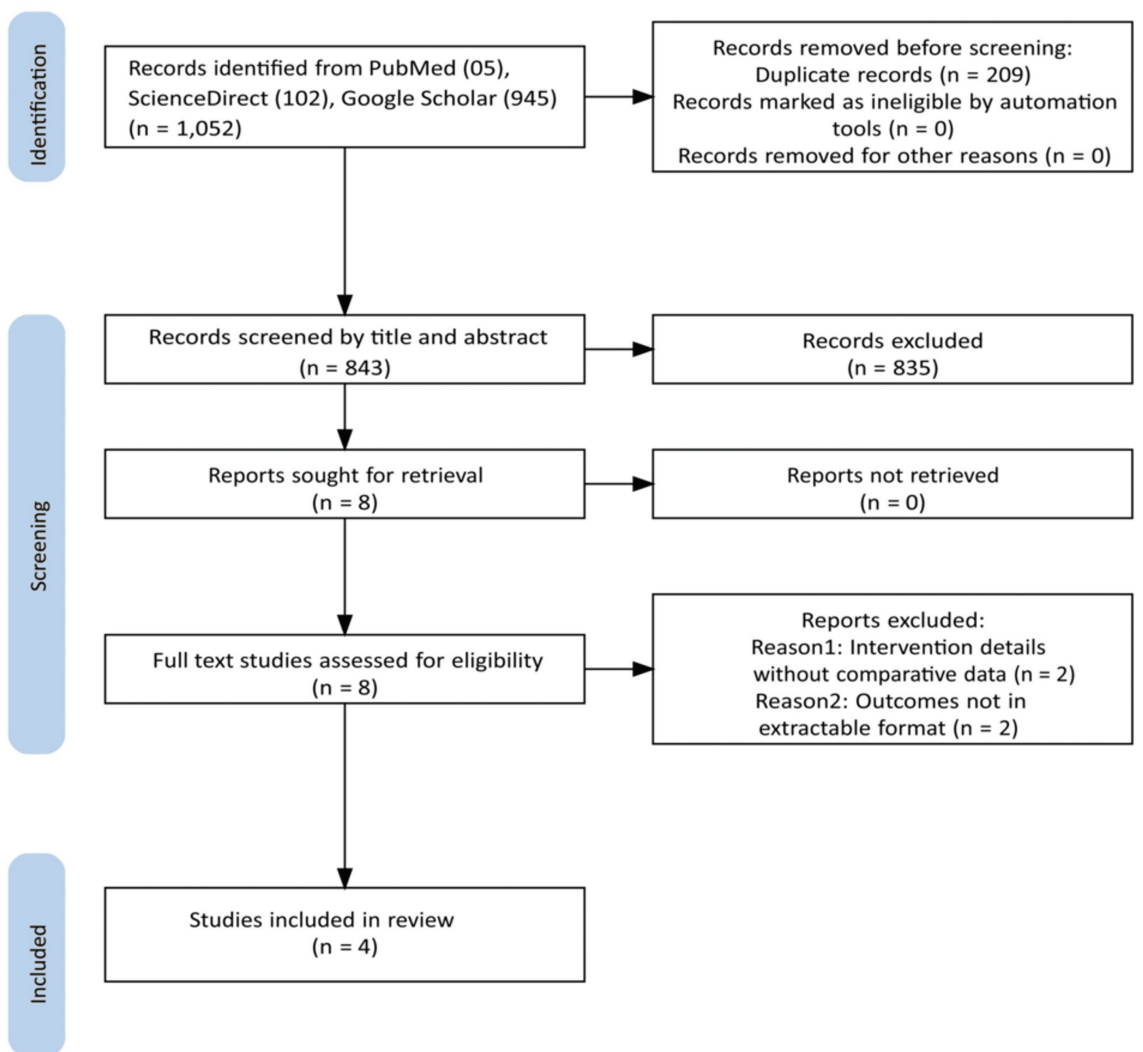
The PRISMA flowchart. PRISMA, Preferred Reporting Items for Systematic Reviews and Meta-Analyses [21].

This meta-analysis was conducted in accordance with the Preferred Reporting Items for Systematic Reviews and Meta-Analyses (PRISMA) guidelines, as shown in Figure 1 [21]. The current project was prospectively registered with the Open Science Framework on March 23, 2025 (https://doi.org/10.17605/OSF.IO/G2H8X), before any data collection.

The patient/population, intervention, comparison, and outcomes (PICO) framework consisted of perinatal women as the population, CBT-I as the intervention, and any non-CBT-I therapy for sleep issues as the comparison. The primary outcome was postpartum depression, while the secondary outcomes were insomnia severity and total sleep time.

A literature search was performed in MEDLINE (via PubMed), ScienceDirect, and Google Scholar up to March 21, 2025. The search was done using query (CBTI OR "Cognitive Behavioral Therapy for Insomnia" OR "CBT-I") AND ("Postpartum Depression" OR "Perinatal Depression" OR "Maternal Depression"). No restrictions were applied regarding date of publication or language. Inclusion criteria were perinatal population, CBT-I as the intervention, and comparison with any other treatment for insomnia. Exclusion criteria included non-human participants, non-RCTs, and studies with interventions other than CBT-I.

All records identified were imported into the citation management software EndNote 21 (Clarivate, Philadelphia, PA). Duplicate studies were omitted. All remaining records were screened by title, abstract, and full text. Screening and selection were performed by Z. Ali and Z. Ehsaan, with O. Akhtar consulted to resolve any disputes or disagreements.

All studies deemed eligible for the meta-analysis were thoroughly reviewed by Z. Ehsaan, M. Maaz, and S. Ahmad. Data, including study design, year of publication, number of participants, type of intervention, mean age, follow-up period, and other relevant information, were extracted and entered into Microsoft Excel, and later exported to Review Manager (RevMan, Version 5.4, The Nordic Cochrane Centre, The Cochrane Collaboration, Copenhagen, Denmark, 2020). Data regarding primary and secondary outcomes were also extracted along with baseline characteristics of participants. Disagreements were resolved through mutual discussion with O. Akhtar.

Cochrane Risk of Bias Tool (RoB Tool, version 2) was used to assess the RoB in individual studies, as shown in Table 1 [22]. Two authors, i.e., Z. Ali and O. Akhtar, independently assessed and estimated the RoB in accordance with random sequence generation and allocation concealment, blinding of participants and personnel, blinding of outcome assessment (both self-reported and objective measures, detection bias), incomplete outcome data, and selective reporting. For each domain, low-risk, some concern, and high-risk scores were assigned, and an overall assessment was subsequently performed based on these scores. In cases of conflict or disagreement, consultation with O. Akhtar was sought for resolution.

**Table 1:** List of included studies and their quality assessment. Risk of bias (RoB) assessment of included studies was performed using the Cochrane RoB 2 tool [22], with data from the studies by Kalmbach et al. [23], Verma et al. [24], Manber et al. [25], and Quin et al. [26].

Study ID/Author	Adequate sequence generation (Low/some concerns/High)	Allocation concealment (Low/some concerns/High)	Blinding of participants and personnel (Low/some concerns/High)	Blinding of outcome assessment (Low/some concerns/High)	Incomplete outcome data (Low/some concerns/High)	Selective outcome reporting (Low/some concerns/High)	Free of other bias (Low/some concerns/High)	Overall risk of bias (Low/some concerns/High)
Kalmbach et al., 2020 [23]	Low	Low	Some concerns	Some concerns	Low	Low	Low	Some concerns
Verma et al., 2022 [24]	Low	Low	Some concerns	Some concerns	Low	Low	Low	Some concerns
Manber et al., 2023 [25]	Low	Low	Some concerns	Some concerns	Low	Low	Low	Some concerns
Quin et al., 2024 [26]	Low	Low	Some concerns	Some concerns	Low	Low	Low	Some concerns

Statistical analysis was performed using Review Manager (RevMan, version 5.4). The random effects model was used for the meta-analysis to account for between-studies variance and potential heterogeneity. For continuous outcomes (postpartum depression, insomnia severity, and total sleep time), the mean difference was calculated with a 95% confidence interval.

The I² statistic was used to assess statistical heterogeneity. For sensitivity analysis, each study was sequentially excluded to evaluate its impact on the overall estimate.

Results

Our meta-analysis included four studies with a combined sample size of 381 participants, assessing the efficacy of CBT-I interventions on mental health and sleep outcomes in postpartum patients. Participants had a mean age of 32.08 years, and the majority (269, 70.5%) were White participants. Baseline Insomnia Severity Index (ISI) scores were similar between the groups, with a mean score of 14.38 in the CBT-I group and 14.29 in the control group. Detailed baseline characteristics are presented in Table 2.

**Table 2:** Baseline characteristics of included studies. Baseline characteristics of participants across included studies, with data extracted from Kalmbach et al. [23], Verma et al. [24], Manber et al. [25], and Quin et al. [26]. SD, standard deviation; NA, not available

	Quin 2024		Manber 2023		Verma 2022		Kalmbach 2020	
	Intervention	Control	Intervention	Control	Intervention	Control	Intervention	Control
Sample size	42	41	68	61	39	39	46	45
Age (years), mean ± SD	32.56 ± 3.05	32.93 ± 4.07	33.65 ± 5.03	33.22 ± 4.99	32.85 ± 4.99	31.42 ± 3.77	28.91	29.16
Nulliparous (%)	100	100	58.8	59	100	100	63	66.7
Race/Ethnicity								
White	36	34	41	32	35	34	24	23
Asian	5	7	9	12	3	3	3	3
Black	NA	NA	3	1	-	-	15	14
Others	1	0	14	13	1	2	4	5
Education								
Sub‑Bachelor	6	9	-	-	7	9	-	-
Bachelor	6	14	-	-	17	18	-	-
Postgraduate	28	18	-	-	15	12	-	-
Annual household income								
Under $52,000	1	0	-	-	3	0	-	-
$52,000 to $77,999	0	0	-	-	2	3	-	-
$78,000 to $103,999	3	2	-	-	9	10	-	-
$104,000 to $129,999	4	9	-	-	4	12	-	-
$130,000 to $155,999	12	7	-	-	5	6	-	-
$156,000 and over	23	20	-	-	14	8	-	-
Insomnia Severity Index (ISI) Score, mean ± SD	14.07 ± 4.09	13.83 ± 3.51	15.40 ± 4.32	15.52 ± 4.55	13.15 ± 3.85	13.72 ± 4.54	14.91 ± 3.55	14.07 ± 3.37
Edinburgh Postnatal Depression Scale (EPDS) Score, , mean ± SD	-	-	7.71 ± 4.30	9.18 ± 4.28	NA	NA	7.46 ± 4.14	9.47 ± 4.62
Insomnia disorder (%)	66.7	63.4	-	-	64.1	87.2	-	-
Patient-Reported Health Status for Physical, Mental, and Social Well-Being (PROMIS) Score, mean ± SD	49.97 ± 5.32	51.21 ± 6.68	NA	NA	52.96 ± 6.44	53.41 ± 6.61	NA	NA

Postpartum Depression

**Figure 2:**
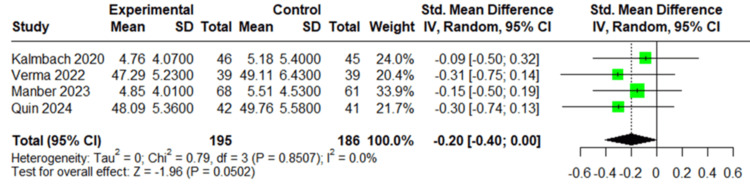
Forest plot of postpartum depression. Forest plot of postpartum depression outcomes using data from Kalmbach et al. [23], Verma et al. [24], Manber et al. [25], and Quin et al. [26].

All four studies (381 patients) reported postpartum depression. The pooled analysis showed borderline significant decrease in postpartum depression scores in the CBT-I group compared to the control group, with a standardized mean difference of -0.20 (95% CI: -0.40 to 0.00; *P* = 0.0502) and no observed heterogeneity (*I*² = 0%), as shown in Figure 2.

Insomnia Severity

**Figure 3:**
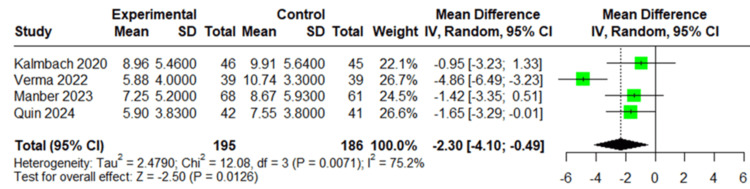
Forest plot of insomnia severity. Forest plot of insomnia severity based on data from the studies by Kalmbach et al. [23], Verma et al. [24], Manber et al. [25], and Quin et al. [26].

**Figure 4:**
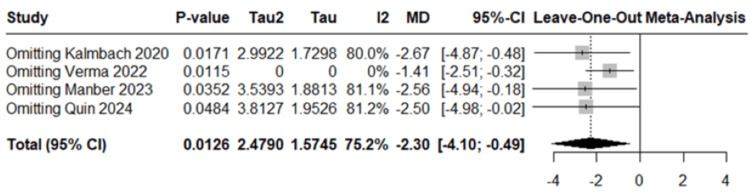
Sensitivity analysis of insomnia severity. Sensitivity analysis of insomnia severity using data from the studies by Kalmbach et al. [23], Verma et al. [24], Manber et al. [25], and Quin et al. [26].

All four studies (381 patients) reported insomnia severity. The pooled analysis showed a statistically significant mean difference of -2.30 (95% CI: -4.10 to -0.49, *P* = 0.0126) favoring the CBT-I group, with substantial heterogeneity (*I*² = 75.2%), as shown in Figure 3. Sensitivity analysis showed that the removal of the study by Verma et al. reduced heterogeneity from 75.2% to 0%, with a mean difference of -1.41 (95% CI: -2.51 to -0.32; *P* = 0.0115), confirming a statistically significant effect and indicating that the initial significant effect was largely driven by this study (Figure 4).

Total Sleep Time

**Figure 5:**
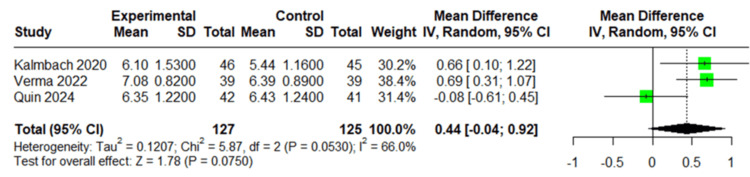
Forest plot of total sleep time. Forest plot of total sleep time using data from the studies by Kalmbach et al. [23], Verma et al. [24], and Quin et al. [26].

**Figure 6:**
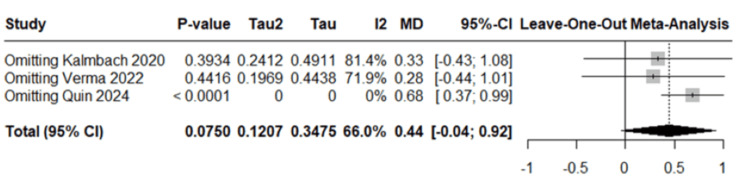
Sensitivity analysis of total sleep time. Sensitivity analysis of total sleep time using data from the studies by Kalmbach et al. [23], Verma et al. [24], and Quin et al. [26].

Total sleep time, assessed through sleep diaries, was reported by three studies with a total of 252 participants. The pooled analysis revealed nonsignificant results, with a mean difference of 0.44 hours (95% CI: -0.04 to 0.92; *P* = 0.075) favoring the intervention group, as shown in Figure 5. Moderate heterogeneity was observed (*I*² = 66%), suggesting inconsistency across the included studies. After sensitivity analysis excluding the study by Quin et al., heterogeneity dropped to zero (*I*² = 0%), and the mean difference increased to 0.68 hours (95% CI: 0.37-0.99), reaching statistical significance (*P* < 0.0001), as shown in Figure 6. This indicates a significant increase in total sleep time favoring the intervention group when the outlier study was removed.

Discussion

Sleep problems are commonly experienced by many mothers both during the pregnancy and after birth. Multiple mechanisms exist to cause this including hormonal variations, infant care demands, and disruption of the body’s natural circadian rhythm. Building on, the risk for postpartum depression is increased by this poor sleep. Biological pathways to cause this are multiple. These include increased inflammatory mediators (higher IL-6 and TNF-α levels), decreased estrogen and progesterone that affect serotonin and GABA systems (sleep neurotransmitters), and changes in stress hormone regulation through the hypothalamic-pituitary-adrenal axis [27-29]. Furthermore, psychological factors also play a major role, including unhelpful ruminations about sleep, spending excessive time in bed beyond sleep hours, and nighttime mental overactivity. Cognitive restructuring, sleep restriction, and stimulus control can be effective in addressing these factors and can be achieved through CBT-I [24,30].

CBT-I is the first-line treatment for chronic insomnia. In postpartum women, it tackles unique challenges such as hormonal changes, nighttime infant care, and relatively higher anxiety and depression rates [9,29]. Studies show that both in-person and digital CBT-I produce significant improvements in sleep that may last up to a year after birth [26,31]. These effect sizes are considerable; however, no data are available for direct comparisons with those observed in the general population. Gaps in the literature remain due to differences in measured outcomes, very limited long-term follow-up, and poor reporting quality in some studies [32,33]. This meta-analysis strengthens the available evidence by providing pooled estimates for postpartum women. Thus, aiming to help address the lack of insomnia-focused integrated evidence in this group.

The prevalence of postpartum depression is approximately 13%-19% among women in the first six months after birth. Additionally, sleep disturbances are a nearly universal experience during this period [34]. Major guidelines, including those from the United States Preventive Services Task Force (USPSTF), the National Institute for Health and Care Excellence (NICE), the American Psychiatric Association (APA), and the World Health Organization (WHO), recommend CBT as a first-line treatment for mild to moderate perinatal depression and anxiety. This preference of psychotherapy over medication is to avoid fetal exposure to antidepressants [35,36]. While CBT-I is recognized as effective for postpartum sleep problems, there are no available clinical guidelines that provides detailed and specified use in these women [37,38]. Clinical applicability is also limited by poor screening adherence, a lack of trained therapy providers, population-level stigma, and systemic barriers, particularly among low-income women [19,20]. This meta-analysis addresses this issue by pooling available data on both sleep and mood outcomes, thereby providing evidence that may support the development of guidelines for CBT-I in maternal healthcare.

RCTs of CBT-I in postpartum women face major methodological challenges. Most trials have small sample sizes, very limited blinding, and incoherent inclusion criteria [32,39,40]. Participants are often demographically narrow, and intervention fidelity is rarely assessed. All this makes it unclear whether studies truly tested comparable CBT-I programs [9,41]. Furthermore, outcome measured vary widely. This forces researchers to rely on standardized mean differences and complicates clinical interpretation [42]. These limitations reduce the reliability and generalizability of the available evidence. There is growing need for larger, well-reported, and culturally diverse trials with consistent methodology to assess clinical applicability.

Postpartum insomnia has strong association with depression and exhaustion affecting mother-infant bonding and infant development [11,43]. CBT-I improves sleep and alongside depressive symptoms, anxiety, and fatigue. These benefits are observed from mid-pregnancy through the postpartum period [26,44,45]. Most trials show sustained small-to-medium mood betterment. Others have reported limited effects on depression despite improved sleep [24]. Theory suggests that better maternal sleep could enhance parenting and child development via improved bonding and responsiveness yet measured evidence in this regard is limited. This is because most studies measure only maternal sleep and mood, not commenting directly on parenting. Hence, the evidence for long-term improved child development with CBT-I is indirect. Addressing these gaps could strengthen the case for the integration of CBT-I in the routine perinatal and postpartum care.

Furthermore, the limited number of available studies made subgroup analysis or meta-regression impossible. However, variations in comparator type (e.g., sleep education, pseudo-desensitization, psychoeducation and sleep hygiene information) may have influenced the observed effect sizes. Other potential sources of heterogeneity, include delivery format (digital vs. in‑person), baseline insomnia severity, and postpartum assessment timing. These factors could not be examined statistically but represent important considerations for future research and clinical implementation. In addition to these statistical limitations, this study has limited generalizability due to the predominantly White and high-income sample.

## Conclusions

CBT-I, despite having growing evidence of effectiveness in postpartum depression, remains an underutilized and underutilized clinical option for pregnant women. Multiple reasons can be accounted for behind this discrepancy. These include limited trained personnel, clinicians’ awareness, and general population stigma involving behavioral therapies. Research and evidence gaps remain, as only a limited number of studies have explored cognitive behavioral therapy for insomnia in pregnant women. This meta-analysis aims to help address this gap by providing evidence that may support the integration of this intervention into maternal healthcare services.
